# P-MAPA and Interleukin-12 Reduce Cell Migration/Invasion and Attenuate the Toll-Like Receptor-Mediated Inflammatory Response in Ovarian Cancer SKOV-3 Cells: A Preliminary Study

**DOI:** 10.3390/molecules25010005

**Published:** 2019-12-18

**Authors:** Luiz Antonio Lupi, Flávia Karina Delella, Maira Smaniotto Cucielo, Graziela Gorete Romagnoli, Ramon Kaneno, Iseu da Silva Nunes, Raquel Fantin Domeniconi, Marcelo Martinez, Francisco Eduardo Martinez, Wagner José Fávaro, Luiz Gustavo de Almeida Chuffa

**Affiliations:** 1 Department of Anatomy, UNESP-São Paulo State University, Institute of Biosciences, Botucatu, 18618-689 São Paulo, Brazil; luiz.lupi@unesp.br (L.A.L.); maira.cucielo@gmail.com (M.S.C.); raquel.domeniconi@unesp.br (R.F.D.); fe.martinez@unesp.br (F.E.M.); 2 Department of Morphology, UNESP-São Paulo State University, Institute of Biosciences, Botucatu, 18618-689 São Paulo, Brazil; flavia.delella@unesp.br; 3 Department of Microbiology and Immunology, UNESP-São Paulo State University, Institute of Biosciences, Botucatu, 18618-689 São Paulo, Brazil; graziela.romagnoli@unesp.br (G.G.R.); rskaneno@yahoo.com.br (R.K.); 4 Farmabrasilis R&D Division, Campinas, 13279-020 SP, Brazil; iseununes@gmail.com; 5 Department of Morphology and Pathology, Federal University of São Carlos, 13565-905 São Paulo, Brazil; martinez@ufscar.br; 6 Department of Structural and Functional Biology, UNICAMP-University of Campinas, Campinas, 13083-970 São Paulo, Brazil; favarowj@unicamp.br

**Keywords:** ovarian cancer, P-MAPA, IL-12, TLR signaling, inflammation, chemoresistance

## Abstract

Immunotherapies have emerged as promising complementary treatments for ovarian cancer (OC), but its effective and direct role on OC cells is unclear. This study examined the combinatory effects of the protein aggregate magnesium–ammonium phospholinoleate–palmitoleate anhydride, known as P-MAPA, and the human recombinant interleukin-12 (hrIL-12) on cell migration/invasion, apoptosis, toll-like receptor (TLR)-mediated inflammation, and cytokine/chemokine profile in human OC cell line SKOV-3. P-MAPA and IL-12 showed cancer cell toxicity under low doses after 48 h. Although apoptosis/necrosis and the cell cycle were unchanged by the treatments, P-MAPA enhanced the sensitivity to paclitaxel (PTX) and P-MAPA associated with IL-12 significantly reduced the migratory potential and invasion capacity of SKOV-3 cells. P-MAPA therapy reduced TLR2 immunostaining and the myeloid differentiation factor 88 (MyD88), but not the TLR4 levels. Moreover, the combination of P-MAPA with IL-12 attenuated the levels of MyD88, interferon regulatory factor 3 (IRF3) and nuclear factor kappa B (NF-kB p65). The IL-12 levels were increased and P-MAPA stimulated the secretion of cytokines IL-3, IL-9, IL-10, and chemokines MDC/CCL22 and, regulated on activation, normal T cells expressed and secreted (RANTES)/CCL5. Conversely, combination therapy reduced the levels of IL-3, IL-9, IL-10, MDC/CCL22, and RANTES/CCL5. Collectively, P-MAPA and IL-12 reduce cell dynamics and effectively target the TLR-related downstream molecules, eliciting a protective effect against chemoresistance. P-MAPA also stimulates the secretion of anti-inflammatory molecules, possibly having an immune response in the OC microenvironment.

## 1. Introduction

Ovarian cancer (OC) is the fifth largest cause of cancer-related death in the United States and is the most lethal of all gynecological malignancies [1]. OC originates from the ovarian surface epithelium, fallopian tube (fimbriae region) or endometriosis-related tissue, and often exhibits a late diagnosis due to the lack of clear signs or symptoms in the early stages of development [2,3,4,5]. Unfortunately, there is no screening method for achieving the best curative result, and traditional chemotherapy and surgery are limited for patients with advanced OC [6]. After cisplatin and paclitaxel (PTX) resistance, several patients become susceptible to developing recurrent OC and metastasis [7]. Therefore, new therapeutic options that overcome chemoresistance and enhance drug sensitivity are promising for the treatment of OC.

Immunotherapies have demonstrated great efficiency by activating host immune responses into the OC microenvironment [8]; however, what is happening with the cancer cells as a direct result of immunostimulation requires further investigation. We recently reported the effect of the immunomodulatory agent termed protein aggregate magnesium-ammonium phospholinoleate-palmitoleate anhydride (P-MAPA), a natural biopolymer extracted from the *Aspergillus oryzae*, which exhibits a number of antitumor responses in different experimental models of cancer [9,10,11]. In association with cisplatin, P-MAPA showed a greater survival rate and a reduced OC volume in addition to the increased expression of proteins involved in toll-like receptor (TLR)-mediated inflammatory response (canonical and non-canonical pathways) in OC-bearing animals [11]. Recent studies also supported the immunological mechanism of action of P-MAPA through the activation of TLR2 and TLR4 signaling in both cancer and infections, in addition to regulating the activity of T cells (especially CD4+T and CD8+T cells) and natural killer (NK) cells [9,10]. Importantly, P-MAPA did not show toxicity in preclinical in vitro (V-79 Chinese hamster cell line) and in vivo models (Swiss mice, Wistar rats, and monkeys) nor in human clinical trial phase I [9]; its effects in cancer treatment have been tested in non-muscle invasive urinary bladder cancer [9,10] and in OC [11]. The directive role of P-MAPA on human OC cells, considering its feasibility, sensibility, resistance, and toxicity, has not been explored yet.

TLRs are transmembrane molecules that signal via myeloid differentiation factor 88 (MyD88) or TLR-associated activator of interferon (TRIF) to induce cell proliferation, chemoresistance, and cytokine/chemokine production [12]. Most importantly, TLRs can trigger a different response depending on the cell type (e.g., cancer cell or immune cell), and particularly, TLR4 has been reported to be a precursor of the immune escape of OC cells [13,14]. The crosstalk between the immunoadjuvant and the OC cells targeting the TLR signaling and related cytokine secretion has never been proposed as to their impact and specific response.

Interleukin-12 (IL-12) is a cytokine related to innate and adaptive immunity, being mainly produced by the antigen-presenting cells (APCs), such as B lymphocytes, dendritic cells (DCs), and others [15,16]. It acts on T and NK cells stimulating a cytotoxic CD8+ response and inducing cytokine production, especially interferon-γ (IFN-γ) [15,17]; IL-12 is involved in the differentiation of naïve T cells into a polarized T helper 1 (Th1) immune response. Patients with recurrent OC who underwent IL-12 treatment, in a well-established dosage regimen, showed significant tumor regression [18]; the major challenge involving the treatment with IL-12 is related to the adverse effects due to its high diffusion and toxicity (e.g., lymphopenia and irreversible elevation of transaminases at 600 ng/kg and neutropenia, fatigue, and headache at 300 ng/kg) [19], and a more precise and direct administration may limit their undesirable effects. Notably, a phase II study involving intraperitoneal infusions of recombinant IL-12 in patients with residual disease ≤1 cm showed to be well-tolerated after fist-line therapy for OC-related peritoneal carcinomatosis [19]. Recently, Cohen et al. [16] studied the relationship between membrane-bound IL-12 in tumor cells and the potential to disrupt protumorigenic signaling and tumor outgrowth within peritoneal cavities; IL-12 significantly led to a tumor refractory state, thereby delaying the onset of metastatic disease.

Because P-MAPA is thought to increase IFN-γ levels, which may potentiate the Thelper (Th1)-mediated immune response, and adjuvant therapies with IL-12 have long been proposed as beneficial for patients with OC, this study investigates the effects of P-MAPA and IL-12, alone or in combination, on cancer cell activities with focus to the TLR-mediated inflammatory process and cytokines/chemokines profiling in human SKOV-3 cell line.

## 2. Results

### 2.1. P-MAPA and IL-12 Reduce Cell Viability and Induce Apoptosis in the Presence of PTX in SKOV-3 Cells

**Figure 1:**
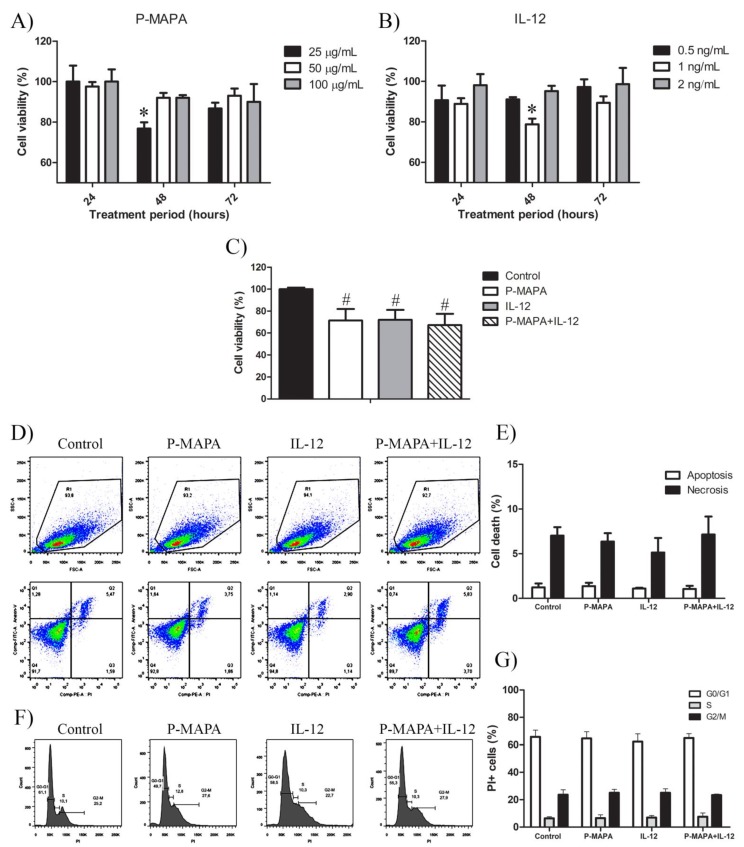
Protein aggregate magnesium–ammonium phospholinoleate–palmitoleate anhydride (P-MAPA) and interleukin-12 (IL-12) suppress cell viability without changing the apoptosis rate and cell cycle. SKOV-3 cells were treated with various concentrations of P-MAPA (**A**) and IL-12 (**B**) for 24, 48, and 72 h, and the cytotoxicity (expressed as percentage) was assayed by MTT; (**C**) Cell viability (%) after standardization of treatments (25 µg P-MAPA and 1 ng IL-12) after 48 h exposure; 1 × 10^3^ SKOV-3 cells showed the best reproducibility. (**D**) Representative apoptotic index in SKOV-3 cells treated with P-MAPA and IL-12 for 48 h detected by annexin V/PI flow cytometry. (**E**) Percentage of cells in apoptosis and necrosis. (**F**) Representative cell cycle analysis in SKOV-3 cells treated with P-MAPA and IL-12 for 48 h detected by PI and RNAase flow cytometry. (**G**) Percentage of PI+ cells in G0/G1, S, and G2/M phases. The samples were assayed in three technical and biological replicates. Results are expressed as the mean ± SD and described as column chart. * *p* < 0.05 vs. different doses at 48 h; # *p* < 0.05 vs. control group.

An MTT assay was carried out using three biological and technical replicates to unravel the most suitable dose and period of treatment. Based on previous results, the SKOV-3 cells were challenged with P-MAPA at doses of 25 µg/mL, 50 µg/mL, and 100 µg/mL, and rhIL-12 at doses of 0.5 ng/mL, 1 ng/mL, and 2 ng/mL for 24 h, 48 h, and 72 h. Then, we tested three different cell concentrations to achieve the better treatment response (1 × 10^3^ cells, 1 × 10^4^ cells, and 5 × 10^4^ cells; Supplementary Figure S1). The SKOV-3 cell viability was efficiently reduced after the low-dose P-MAPA treatment (25 µg/mL for 48 h; viability was reduced by ~27%), and then started to increase after 72 h exposure (Figure 1A). Treatment with rhIL-12 also showed reduction in cell viability at an intermediate dose of 1 ng/mL mainly after 48-h exposure (Figure 1B; viability reduction by ~25% after 48 h). Combinatory therapy with P-MAPA and IL-12 also reduced the cell viability by about 33% (Figure 1C), and the treatment doses were set at 25 µg/mL of P-MAPA and 1 ng/mL of IL-12. Annexin V- fluorescein isothiocyanate (FITC)/Propidium iodide (PI) staining was used to effectively determine the apoptosis/necrosis rate induced by P-MAPA and rhIL-12 immunotherapies. In this study, we set early apoptosis (Annexin V-FITC+/PI−) as apoptosis and late apoptosis (Annexin V+/PI+) and necrosis (Annexin V−/PI+) as necrosis. The apoptosis index was not influenced by P-MAPA, IL-12, or P-MAPA+IL-12 (Figure 1D,E), which pointed out that these agents are unable to induce cell death at this concentration. We also observed that P-MAPA and IL-12 did not alter the cell cycle significantly (Figure 1F,G). These data indicate that these immunotherapeutic agents do not have a direct and high toxic effect to the cell, which is, in fact, expected from immunotherapies. Because the treatments did not promote cell death or cell proliferation, the reduction of cell viability might have been a result of decreased cell metabolism, because the MTT test indirectly reflects the mitochondrial activity.

**Figure 2:**
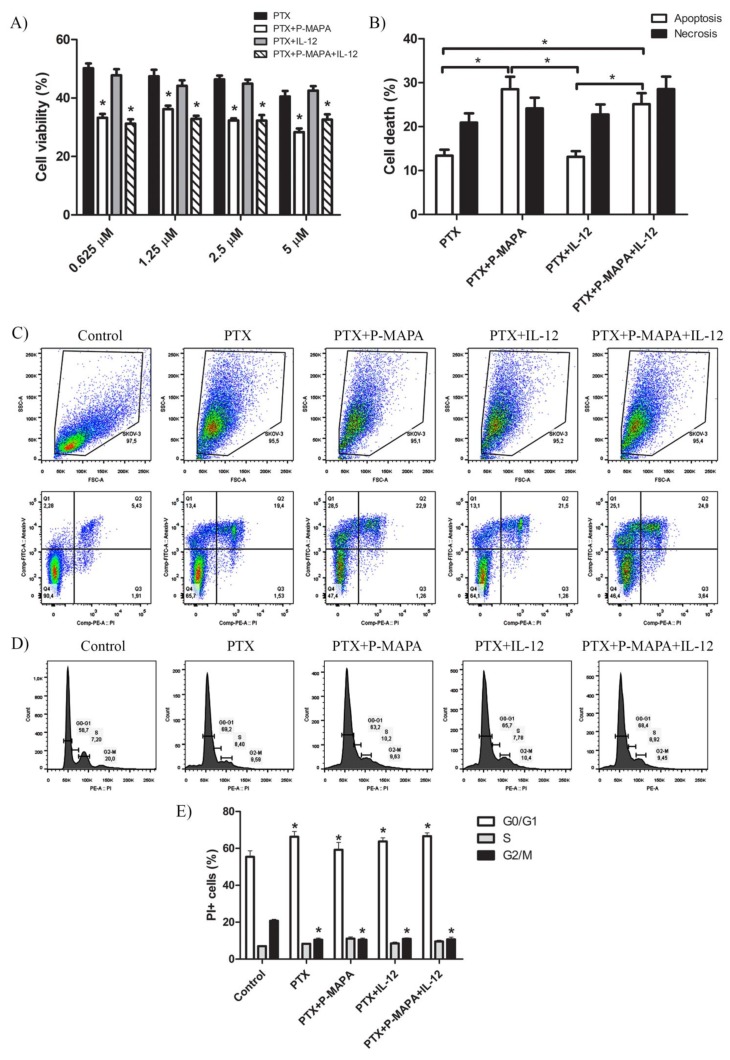
P-MAPA and P-MAPA+IL-12 reduce cell viability and apoptosis/necrosis rate in the presence of PTX. (**A**) Cell viability was assessed by an MTT assay; SKOV-3 cells were treated with various concentrations of PTX alone or in association with P-MAPA and IL-12 for 48 h. * *p* < 0.05 vs. PTX and PTX+IL-12 groups. (**B**) Percentage of cells in apoptosis and necrosis after exposure to 0.625 µM PTX plus P-MAPA, IL-12, or P-MAPA+IL-12. * *p* < 0.05. (**C**) Representative apoptotic index in SKOV-3 cells detected by Annexin V/PI flow cytometry. (**D**) Representative cell cycle analysis in SKOV-3 cells treated with PTX, P-MAPA and IL-12 for 48 h detected by PI and RNase flow cytometry. (**E**) Percentage of PI+ cells in the G0/G1, S, and G2/M phases. The samples were assayed in three technical and biological replicates.* *p* < 0.05 vs. control group. Results are expressed as the mean ± SD.

To better understand whether P-MAPA and IL-12 therapies potentially increase the effect of standard chemotherapy, they were tested in association with PTX (Figure 2A,B). After 48 h, P-MAPA decreased cell viability in association with different doses of PTX compared with cells treated with PTX only (~30% reduction with 5 µM PTX; ~30% reduction with 2.5 µM PTX; ~24% reduction with 1.25 µM PTX and ~34% reduction with 0.625 µM PTX). Because IL-12 had no effect on cell viability when associated with PTX, the decreased cell viability observed after combining P-MAPA with IL-12 in association with doses of PTX is probably due to the P-MAPA effect (~19% reduction with 5 µM PTX, ~30% reduction with 2.5 µM PTX, ~30% reduction with 1.25 µM PTX, and ~38% reduction with 0.625 µM PTX). To confirm these effects on SKOV-3 cell death, an Annexin V-FITC/PI assay was performed using the lowest dose of PTX (0.625 µM). Concordantly, with the MTT results, P-MAPA and P-MAPA+IL-12 increased cell death in association with PTX at dose of 0.625 µM. The apoptosis/necrosis ratio of PTX was 0.64 and became higher when associated with P-MAPA (1.18) or P-MAPA+IL-12 (0.88), thus enhancing the P-MAPA effect as apoptotic inductor. Finally, PTX significantly increased the number of cells in G0/G1 and decreased cells in G2/M phase of the cell cycle, thus inducing cell cycle arrest. In this case, the addition of P-MAPA and IL-12 did not potentiate the PTX effects in cell cycle (Figure 2D,E).

### 2.2. Combination of P-MAPA with IL-12 Is Essential to Reduce Cell Migration Whereas P-MAPA Alone Decreased The Invasive Potential of SKOV-3 Cells

**Figure 3:**
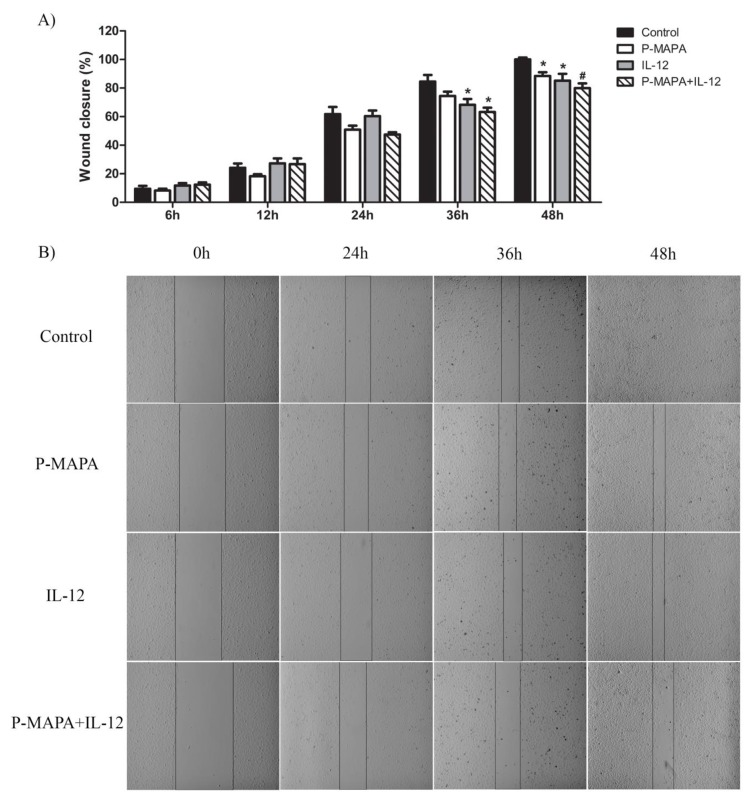
Migratory potential of SKOV-3 cells determined by wound-healing assay. (**A**) Percentage of wound closure after 0, 6, 12, 24, 36, and 48 h. * *p* < 0.05, # *p* < 0.01 vs. control group. (**B**) Photographs of each wound-healing analysis at 0, 24, 36, and 48 h which were representative for specific closing area; vertical black bars were used to show the incision edges (10× magnification). The samples were assayed in three technical and biological replicates.

**Figure 4:**
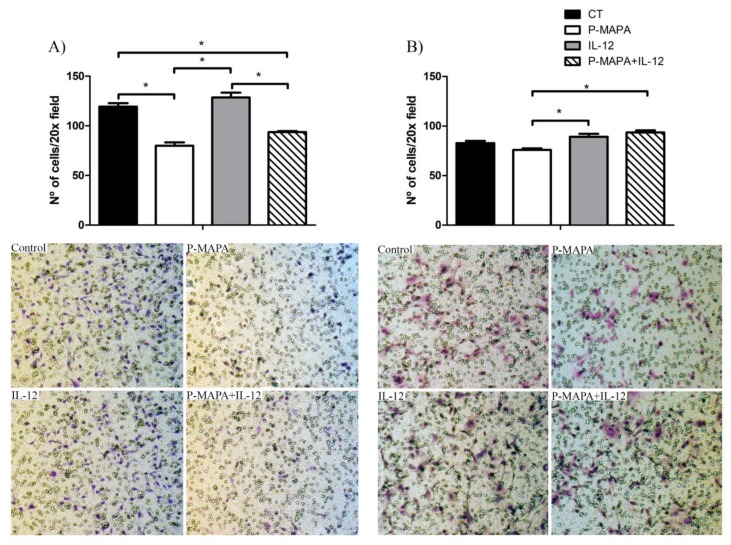
Effects of P-MAPA and IL-12 alone or in combination on the migration and invasion capacity of SKOV-3 cells. (**A**) Cell migration was measured by the amount of cells located in the lower part of the insert. (**B**) Cell invasion was determined by the amount of cells located in the lower part of the insert previously covered by Geltrex^®^. Representative images of the assay were obtained at 20× magnification. The samples were assayed in three technical and biological replicates. Results are expressed as the mean ± SD; * *p* < 0.05; CT, control group.

To investigate the inhibitory effect of P-MAPA and IL-12 as a single or combinatory treatment on SKOV-3 cells, a wound-healing assay was performed in different periods. Although the treatment with IL-12 showed a reduced migration rate (~ 17%) after 36 h and 48 h exposure, cells treated with P-MAPA migrated significantly less (~ 13%) only at 48 h exposure (Figure 3A,B). Combination of P-MAPA and IL-12 reduced the migratory potential of cells after 36 h and 48 h treatment (>20%), being the most efficient after 36 h (Figure 3A,B); this overall analysis suggests that IL-12 was more effective than P-MAPA in delaying wound closure. SKOV-3 cells that were treated with 0.9% saline solution (control group) had an accelerated growth and migration rate when compared with all the treatments. Because the wound-healing assay might be biased by cell proliferation, transwell migration and invasion assays were performed and effectively showed that P-MAPA reduced cell migration when administered alone or in association with IL-12 (Figure 4A). When Geltrex^®^ was added to the chambers, the number of invasive cells was reduced after P-MAPA treatment in comparison to IL-12 treatment or its association (Figure 4B).

### 2.3. Immunotherapy with P-MAPA and IL-12 Significantly Reduced the TLR-Mediated Downstream Molecules Involved in the Inflammatory Process of SKOV-3 Cells

**Figure 5:**
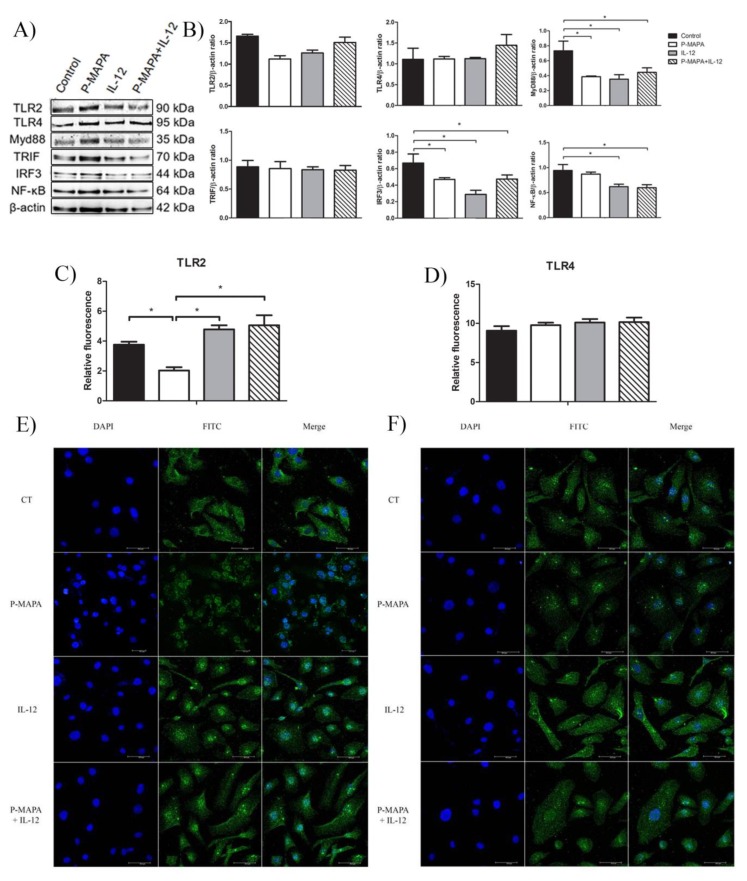
TLR2- and TLR4-mediated signaling pathways are involved with inflammatory process and chemoresistance in human OC. (**A**) Representative protein profiles of TLR2, TLR4, MyD88, TRIF, IRF3, and NF-kB; fractions containing 50 µg protein were pooled from 5 samples per group. (**B**) Individual blots were used for densitometric analysis of the TLR2 and TLR4 levels and related downstream molecules (MyD88, TRIF, IRF3, and NF-kB) after normalization to the β-actin. Data are expressed as the mean ± SD. * *p* < 0.05 vs. control group. Relative fluorescence intensity of TLR2 (**C**) and TLR4 (**D**) receptors. The values are expressed as the mean ± SD. The samples were assayed in three technical and biological replicates. * *p* < 0.05. Confocal imaging of TLR2 (**E**) and TLR4 (**F**) immunostaining using fluorescein isothiocyanate (FITC)-conjugated antibodies anti-TLR2 and anti-TLR4 was obtained in SKOV-3 cells (Alexafluor^®^488, bar = 50 µm). DAPI was used for nuclear staining and merged images were performed using Image J software. Negative controls were used. CT, control; DAPI, 4′,6-diamidino-2-phenylindole; FITC, fluorescein isothiocyanate.

One of the most important factors responsible for the acquisition of malignant phenotypes of OC cells is associated with chemoresistance to treatments and uncovering new agents that downregulate the inflammatory pathway(s) may be of significant interest. To evaluate whether P-MAPA or IL-12 play a role on OC-related inflammation, TLR2- and TLR4-mediated pathways were evaluated through canonical and non-canonical signaling. Although the expressions of TLR2 and TLR4 did not vary significantly, the downstream target molecules were affected by the treatments (Figure 5A,B). P-MAPA and IL-12 alone or in combination led to a profound reduction in the MyD88 levels (0.53-, 0.48-, and 0.61-fold reduction, respectively vs. the control group; Figure 5A,B). We also evaluated the NF-kB p65 expression in the extracts of SKOV-3 cells, and notably, IL-12 alone or combined with P-MAPA induced a significant reduction in the p65 levels (0.66- and 0.63-fold reduction, respectively vs. control group; Figure 5A,B). To explore the non-canonical pathway, the TRIF and IRF3 levels were measured. Although the TRIF levels were unchanged after the treatments (*p* > 0.05), P-MAPA, IL-12, and the combination of P-MAPA with IL-12 induced a significant decrease in the IRF3 levels (0.70-, 0.43-, and 0.71-fold reduction, respectively vs. the control group; Figure 5A,B). These findings provide evidence that P-MAPA and IL-12 potentially act on MyD88-dependent and MyD88-independent pathways, which also encourages the possibility that these immunomodulatory agents could even increase the chemosensitivity of other therapeutics.

To further investigate and elucidate the location and the relative expression levels of the TLR2 and TLR4 receptors, an immunofluorescence assay was performed on SKOV-3 cells. As evidenced by the cellular fluorescence level, the P-MAPA treatment significantly decreased the expression level of cytoplasmic and nuclear TLR2 (48% fluorescence reduced vs. the control group; Figure 5C). On the contrary, IL-12 and the combination of P-MAPA and IL-12 promoted the highest TLR2 immunofluorescence intensity (135% and 151% fluorescence level, respectively vs. P-MAPA; Figure 5C), which suggests that IL-12 is responsible for restoring TLR2 activation after P-MAPA therapy in SKOV-3 cells. Lastly, the relative immunofluorescence of the TLR4 was unchanged after the treatments (Figure 5D).

### 2.4. P-MAPA Stimulates the Secretion of Pro- and Anti-Inflammatory Molecules, Whereas Its Association with IL-12 Induced the Synthesis of Inflammatory Cytokines in SKOV-3 Cells

**Figure 6:**
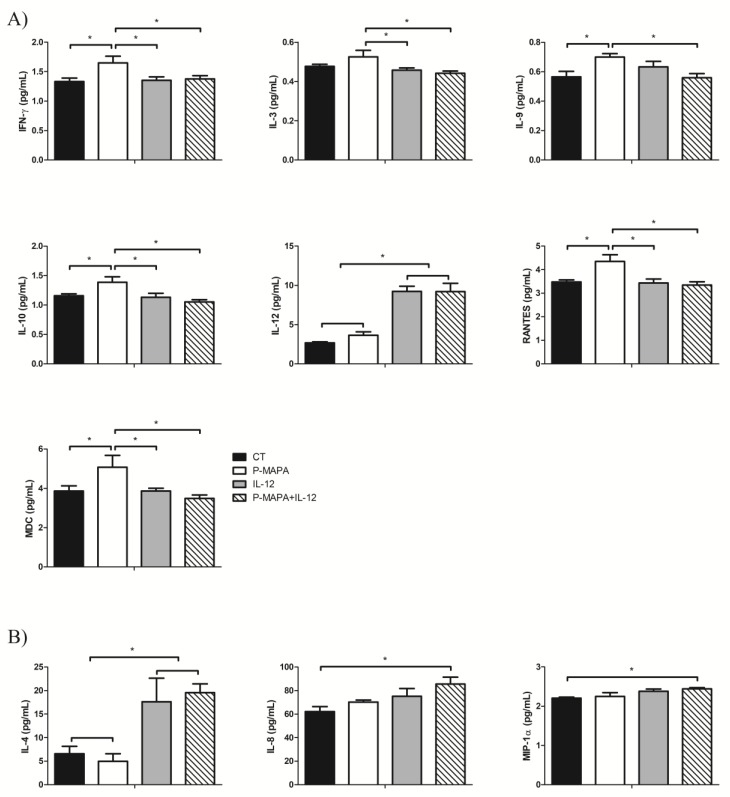
Multiplex analysis of cytokines and chemokines produced by SKOV-3 cells in response to P-MAPA and IL-12 treatments. (**A**) Concentrations of IFN-γ, IL-3, IL-9, IL-10, IL-12, RANTES, and MDC were altered in the supernatants of cell culture. (**B**) Levels of IL-4, IL-8, and MIP-1α were altered in SKOV-3 cell extracts. All data are expressed as the mean ± SD. * *p* < 0.05 as compared with the corresponding group; One-way ANOVA complemented by the Tukey test. The samples were assayed in three technical and biological replicates. CT, control; IFN-γ, interferon gamma; RANTES, regulated on activation, normal T cell expressed and secreted; MDC, macrophage-derived chemokine; MIP-1α, macrophage inflammatory protein 1-alpha.

To determine which cytokines/chemokines are secreted by the SKOV-3 cells and how P-MAPA and IL-12 act to regulate its production, a wide range of these molecules were evaluated in both supernatants (Figure 6A) and cellular extracts (Figure 6B). As expected, the IL-12 levels were higher in the cells treated with IL-12 and P-MAPA+IL-12 (244% and 243% fold-increased, respectively vs. the control group, and 154% and 155% fold-increased, respectively vs. the P-MAPA group; Figure 6A); IL-12 was slightly elevated with P-MAPA, even at low concentrations.

Comparing the treatments, P-MAPA significantly stimulated the secretion of IFN-γ (24% vs. the control, IL-12 and P-MAPA+IL-12 groups; Figure 6A), which is probably related to the activation of the Th1 response. P-MAPA also stimulated IL-10 (21% vs. control, 23% vs. IL-12 and 32% vs. P-MAPA+IL-12 groups), MDC/CCL22 (31% vs. control and IL-12 groups, and 45% vs. P-MAPA+IL-12), and, regulated on activation, normal T cells expressed and secreted (RANTES)/CCL5 (25% vs. the control, 27% vs. the IL-12, and 30% vs. the P-MAPA+IL-12 groups). Furthermore, P-MAPA increased IL-3 and treatment with IL-12 or P-MAPA+IL-12 promoted a reduction in its levels (13% and 17% reduction, respectively vs. P-MAPA; Figure 6A). Treatment with P-MAPA also increased the IL-9 levels (25% increased vs. the control and P-MAPA+IL-12 groups; Figure 6A). A part of these results suggests the secretion of molecules involved in the Th2 response (e.g., IL-10, CCL22), but their regulatory signaling combined with other molecules released by immune cells into the OC microenvironment could not be proved to have a favorable or unfavorable effect. The IL-4 levels were internally elevated in SKOV-3 cells (Figure 6B) after therapies with IL-12 alone or combined with P-MAPA (169% and 254% increased vs. the control group and 198% and 293% increased vs. the P-MAPA group, respectively). Interestingly, the association of P-MAPA with IL-12 induced the highest production of intracellular IL-8 (37% vs. the control group) and MIP-1α (11% vs. control group; Figure 6B). In brief, cytokines that were not significantly influenced by P-MAPA and IL-12 included IL-1β, IL-2, IL-6, IL-7, IL-13, IL-15, IL-17, IP-10, MCP-1, and MIP-1β (Supplementary Tables S1 and S2).

## 3. Discussion

**Figure 7:**
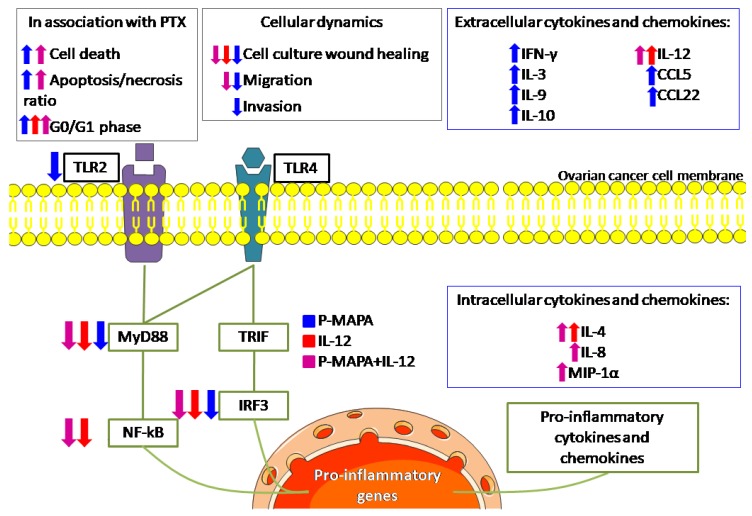
Schematic view of the effects of P-MAPA and IL-12 on human SKOV-3 cells. The effect of each treatment is shown by its representative color. Up and down arrows indicate whether the treatment increased or decreased, respectively, such as cellular function or molecule at these levels. The treatments reduced the downstream molecules (MyD88, NF-kB, and IRF3, with the exception of P-MAPA in reducing NF-kB) of both canonical and non-canonical toll-like receptor (TLR) signaling pathways, and only P-MAPA significantly downregulated the TLR2 levels. The intracellular cytokine/chemokine levels were increased after treatment with IL-12 (IL-4) or P-MAPA+IL-12 (IL-4, IL-8 and MIP-1α); P-MAPA was more effective in increasing the secretion of the following cytokines/chemokines: IFN-γ, IL-3, IL-9, IL-10, RANTES/CCL5, MDC/CCL22). As expected, IL-12 was used to treat SKOV-3 cells and appeared increased after IL-12 and P-MAPA+IL-12 treatments. Although IL-12 only reduced the SKOV-3 cells’ wound closure, P-MAPA efficiently regulated the cellular dynamics by reducing the wound closure, cell migration and invasion. The combination of P-MAPA with IL-12 decreased the period of wound closure and cell migration rate. MyD88, myeloid differentiation primary response 88; NF-kB, nuclear factor kappa B; TRIF, TIR-domain-containing adapter-inducing interferon-β; IRF-3, interferon regulatory factor 3; TLR2, toll-like receptor 2; TLR4, toll-like receptor 4; P-MAPA, protein aggregate magnesium-ammonium phospholinoleate-palmitoleate anhydride; IL, interleukin; MIP-1α, macrophage inflammatory protein 1α; IFN-γ, interferon gamma; RANTES, regulated on activation, normal T cell expressed and secreted; MDC, macrophage-derived chemokine; CCL, C-C motif chemokine ligand.

We reported that lower doses of P-MAPA efficiently reduce cell invasion, whereas P-MAPA in combination with IL-12 reduces cell migration in addition to attenuating the TLR-mediated inflammatory response in human SKOV-3 cells (Figure 7). Although these compounds have individually showed important effects in reducing OC volume and mass while enhancing overall survival and immunostimulation in animals and humans [11,16], this study is the first to describe a therapeutic rationale against human OC, thus revealing the mechanisms underlying cancer cell-related inflammatory aspects rather than those of immune cells.

P-MAPA and IL-12 elicited a decrease in cell metabolism, but no effect on cell apoptosis/necrosis and cell cycle was observed. Although an MTT assay is largely used as the viability/toxicity assay, it could be biased by decreased metabolism activity. This utilizes mitochondrial machinery to convert a colorless tetrazolium salt solution into purple formazan crystals [20]. Once we had pursued a treatment dose with low cell toxicity but considerable modulatory potential, we believed that either P-MAPA or IL-12 may be changing OC cell metabolism to a reduced activity (metabolomics could be of significant value to find the targets more precisely). The impact of this reduction was indeed confirmed by the migration and invasion assays. P-MAPA was able to reduce the migration and invasion capacity, and the delayed wound closure in IL-12-treated cells may be likely due to its decreased metabolic activity. Given that lower doses of P-MAPA and IL-12 are recommended to be well-tolerated, OC cell apoptosis could be prevented by the low-level toxicity of chemicals; conversely, these concentrations are safely used to activate immune responses and modulate cell metabolism. When associated with PTX, P-MAPA was able to increase cell death and apoptosis/necrosis ratio without potentiating the PTX effects on cell cycle. In accordance with a previous study in which approximately 50% of SKOV-3 cells died after 48 h exposure to 5 µM of PTX [21], we observed that P-MAPA not only was effective to increase cell death in the presence of PTX but also to maintain cell toxicity even after considerably lower levels of PTX. This increase in cell death might be a result of apoptosis induction, since the apoptosis/necrosis ratio was significantly higher in animals treated with the combination of P-MAPA and PTX. Because apoptosis lead to the release of the damage-associated molecular pattern (DAMP), this higher ratio may facilitate immunogenic cell death (ICD) [22].

The pro-inflammatory actions of TLR signaling in cancer cells and immune cells may severely affect tumor progression [23], and, particularly, the immunosuppressive OC microenvironment needs to be continuously immunostimulated [23,24]. Although TLR4 was unchanged by the treatments, P-MAPA induces a reduction in the TLR2 levels together with downstream molecules in SKOV-3 cells. Additionally, we found TLR2 nuclear expression in SKOV-3 cells treated with P-MAPA. Although we are not the first to observe nuclear staining of TLR2 and TLR4 [25], the effect of the switch of cytoplasmic to nuclear expression in SKOV-3 cells remains unclear. Based on our results, this nuclear expression seems not to have any negative effect favoring cancer development. Although the TLR2-induced MyD88-dependent pathway is related to an increase in IL-12 secretion [26], the immunoregulatory mechanisms whereby IL-12 restores TLR2 to levels close to control remain to be elucidated. In immune cells, P-MAPA has reportedly been described as a TLR4 agonist [9,10,11], thereby enhancing the synthesis of cytokines and activating a Th1-polarized response. Our data evidenced the protective effect of P-MAPA in attenuating TLR2-mediated signaling in OC cells. The activation of TLR, especially via MyD88, is associated with increased tumor growth, chemoresistance, and the early recurrence of ovarian epithelial tumors [7,27]. In addition, OC expressing high levels of MyD88 presents a higher proliferative index and increased production of pro-inflammatory cytokines and chemokines [7,28,29]. Importantly, the association of P-MAPA and IL-12 decreased the levels of MyD88 in SKOV-3 cells compared with their respective control; because P-MAPA and P-MAPA + IL-12 also significantly decreased the migratory and invasive potential of the cells, it can be suggested that the MyD88-dependent signaling pathway may be one of the mechanisms by which SKOV-3 cells promote migration and invasion. It has also been reported that increased expression of MyD88 is related to a decreased sensitivity to chemotherapy (e.g., PTX) in the MyD88-negative OC A2780 cell line [30]; moreover, the activation of MyD88 and NF-kB in MyD88-positive SKOV-3 cells promoted cell proliferation and tumor growth, likely due to the increased secretion of pro-inflammatory cytokines, thus rendering the cells PTX resistant [13]. In this line, the downregulation of MyD88 and NF-kB may be a possible mechanism by which P-MAPA improves the chemosensitivity of PTX in SKOV-3 cells.

The activation of TLR4/MyD88/NF-kB signaling by ligands is strongly related to an inflammatory microenvironment, thereby contributing to a more aggressive OC phenotype and poorer clinical outcomes in women [31]. In SKOV-3 cells, silencing the membrane-associated RING-CH (MARCH), an ubiquitin ligase that downregulates MHC class II expression, resulted in reduced cell migration and invasion in addition to inhibition of NF-kB signaling [32], thus reinforcing that NF-kB may play a role in the regulation of numerous cellular dynamics. Taken together, the combination of P-MAPA and IL-12 was efficient to induce the downregulation of both MyD88 and NF-kB, which can be of great value to enhance the overall survival of patients while attenuating OC progression and metastasis.

TRIF activation is responsible for triggering the TLR non-canonical pathway, thus activating several transcriptional factors, such as NF-kB, IRF3 and AP-1, and resulting in cytokines and type I IFN production [33,34]. Although few studies have explored the role of TRIF/IRF3 in OC cells, they seem to be critical for therapy [35]. Recently, Chuffa et al. [23] reported the downregulation of TRIF and IRF3 in OC-induced rats, whereby the immunomodulatory agent dramatically reduced the volume and mass of ovarian tumors. Importantly, P-MAPA and IL-12, either alone or in association, significantly downregulated IRF3 levels, evidencing the potential of immunotherapies to act by both canonical and non-canonical TLR pathways.

Immunostimulatory and immunosuppressive molecules can contribute to either inhibiting or enhancing anti-tumor immune activity of such a response by immunotherapy. We screened a number of molecules displaying important effects in OC, but most of them displayed no changes in SKOV-3 cells after P-MAPA and IL-12 treatments. Treatment of SKOV-3 with rhIL-12 showed its availability in both IL-12 and P-MAPA+IL-12 groups, thus proving that the agent was present in the supernatants; whether IL-12 therapy was still capable of stimulating more IL-12 production by the cells is uncertain. IL-3 levels are stimulated by P-MAPA therapy, and this increase might be protective for patients during OC chemotherapy; administration of rhIL-3 to patients with platelet count < 75,000/mm^3^ is effective to fight thrombocytopenia and neutropenia after chemotherapy [36]. Moreover, IL-3 is able to intensify the dose of carboplatin for primary advanced OC [37]. P-MAPA also enhanced IL-9 secretion by SKOV-3 cells, and this effect seems to have a dual impact on the immune system. IL-9 was initially recognized as a T-cell growth factor with oncogenic potential. However, Th9 cell-secreted IL-9 has been revisited for the immunity of some tumors [38]. IL-9 activates innate immune cells like mast cells, contributing to tumor growth prevention [38], and in addition to Th9 cell-derived IL-3, induction of adaptive anti-cancer responses that favor DCs survival has been reported in various cancers [39].

Regulatory T (Treg) cells can infiltrate into solid OC or ascitic fluid, contributing to an immunosuppressive microenvironment by secreting IL-10 and TGFB-1 while reducing the IFN-γ levels [40]. Because these cytokines participate either in stimulating or inhibiting the activities of other immune cells, P-MAPA seems to work as a double-edge sword regarding the SKOV-3 cells. Although mechanistically unclear, P-MAPA contributes partially to reducing the immunosuppression, likely due to the increased levels of IFN-γ rather than IL-10. In fact, high IFN-γ secretion meets our proposal by enhancing OC-cell immunogenicity through the recruitment of CD8+T and natural killer (NK) cells, in addition to increasing the anti-tumor activity of macrophages; we recently found that P-MAPA reduces Treg cells and stimulates CD8+T effector cells in OC-bearing animals (unpublished data). Recently, the expression of programmed death-ligand 1 (PD-L1) on tumor cells represents an important pathway by which malignant cells evade the immune system. In SKOV-3 cells, PD-L1 was variably found in the surface and cytoplasm [41], and its expression was correlated with high levels of TNF-α, IL-10, and IL-6 released from tumor-associated macrophages (TAMs). Because P-MAPA and IL-12 attenuated the downstream mediators of the TLR signaling, the altered expression of some cytokines may be independent of TLR-mediated inflammatory response.

A complex network represented by chemokines and its receptors, growth factors, inflammatory products, and other molecules (e.g., NF-kB), is responsible for tumor progression or rejection. Chemokines signal not only for tumor cells but may control tumor development through activation of specific receptors expressed in a variety of cells, thereby regulating the traffic of infiltrating macrophages, lymphocytes, DCs, and neutrophils [42]. The secretion of CCL5, which binds to the CCR4 receptor, is strongly associated with the presence of tumor-infiltrating CD8+T cells; the upregulation of its receptor in activated vaccine-primed T cells improved tumor homing in OC [43]. In SKOV-3 cells, the levels of CCL22/MDC and CCL5/RANTES were higher after P-MAPA therapy. Although these chemokines appear to be associated with a poor prognosis when released by immune cells, the functional meaning of their production by OC cells as to their significance in the OC microenvironment remains to be investigated. CCL22 is secreted by DCs and macrophages and acts on target cells by interacting with the CCR4 receptor located in the cell surface [44]. CCL22 is correlated to the chemoattraction of Treg cells in advanced stages of OC, and its expression was increased in response to IFN-γ signaling [45]. This finding partially corroborates our results in which both CCL22 and IFN-γ were higher in SKOV-3 cells after P-MAPA therapy; in contrast, P-MAPA does not promote elevation in the number of Treg cells (data not shown). A previous study by Giuntoli et al. [46] showed that ascites specimens originating from patients with malignant OC were accomplished by elevated levels of IL-6, IL-8, IL-10, IL-15, IP-10, MCP-1, MIP-1β, and VEGF, in contrast to significantly reduced levels of IL-2, IL-5, IL-7, IL-17, and CCL5/RANTES. Although controversial, IL-8 production by human OC cells plays a role in controlling tumor growth [47], and MIP-1α is involved in the recruitment of Th1 and cytotoxic effector T cells [48]. We found a higher secretion IL-8 and MIP-1α after combinatory treatment of P-MAPA and IL-12. Partially supporting our results, IL-8 levels have already been reported to be augmented in OC, even after the downregulation of NF-kB [49]; however, the alternative mechanism involved with this production remains unclear. Additionally, we observed increased levels of RANTES after P-MAPA treatment. RANTES is responsible for recruiting T cells, macrophages, eosinophils, and basophils into the inflammatory sites. In addition to IL-2 and IFN-γ, RANTES is thought to induce the activation and proliferation of NK cells while enhancing the anti-tumor response in animal models [46]. In addition to the overexpression of VEGF and MCP-1, a reduction in RANTES levels is associated with activating pathways for tumor growth. Normally, OCs produce large amounts of RANTES, and this production is correlated with the infiltration of TAMs, CD8+T cells, and tumor progression [50,51]; these chemokines are further related to the acquisition of polarized immune responses (Th1 versus Th2). Although some negative effects are associated with the efficacy of chemotherapy related to RANTES, therapy with P-MAPA may be helpful against tumor expansion.

## 4. Materials and Methods

### 4.1. Cell Line and Cell Culture

The human OC cell line SKOV-3 was purchased from the American Type Culture Collection (ATCC, Rockville, MD, USA). The SKOV-3 cells were routinely incubated in RPMI 1640 (Life Technologies, Grand Island, NY, USA) supplemented with 10% Fetal Bovine Serum (FBS) and 1% anti-anti solution (100 mg/mL penicillin G, and 100 µg/mL streptomycin (Merck, Darmstadt, Germany). The culture medium was changed every 2 to 3 days. All cells were maintained at 37 °C in a humidified atmosphere of 5% CO_2_.

### 4.2. Treatments with P-MAPA and IL-12

To evaluate the in vitro effect of the treatments, different doses of P-MAPA (25 µg/mL, 50 µg/mL, and 100 µg/mL) were tested in accordance with Favaro et al. [9]. Initially, 5 mg P-MAPA was diluted in 1 mL of saline solution to achieve the desired stock solution of 5 mg/mL, which were then diluted in the cell culture medium to obtain the testing doses. For the treatment with recombinant (rh)IL-12, concentrations of 0.5 ng/mL, 1 ng/mL, and 2 ng/mL were diluted in the culture medium based on the previous report by Su et al. [52]. For the combination of P-MAPA and IL-12, the most effective dose and incubation period were determined after performing an MTT assay. The saline solution was used as a solvent vehicle control and prepared in the same volume and dilution for both treatments. All experiments were performed at 0, 24, 48, and 72 h time exposure and assayed in three technical and biological replicates.

### 4.3. Cell Cytotoxicity (MTT Assay)

The SKOV-3 cells were seeded in a 96-well plate at a density of 1 × 10^3^ cells/well. The cellular activity and/or toxicity were evaluated in different concentrations of P-MAPA and IL-12 and periods (0, 24, and 48 h) to define the best treatment protocol. For this experiment, the choice of dose and period of treatment was the combination of high cellular viability with low toxicity and efficient signaling regulation. Thereafter, different doses of PTX were associated with P-MAPA and IL-12 to verify the sensitivity effects. An MTT solution (5 mg/mL) was added to the wells for 4 h, and the crystals were diluted with DMSO under agitation. The concentration was determined by an Epoch microplate reader (BioTek Instruments, Highland Park, PO, USA) at 540 nm, being the reference curve fixed at 650 nm. The percentage of crystal formation was calculated by fixing the control group crystal formation as 100%.

### 4.4. Apoptosis Rate by Annexin V-FITC/PI Staining

During the apoptosis process, cells normally externalize the phospholipid phosphatidylserine (PS), which binds with high affinity to Annexin V in the presence of calcium. Herein, we used the Annexin V assay with the BD Pharmingen^TM^ Annexin V-FITC Apoptosis Detection Kit (ApoAlert Annexin V, Clontech, CA, USA). The SKOV-3 cells (1 × 10^5^ cells) were placed in a 6-well plate and left for 6 h to attach. The cells were then treated with 25 µg/mL of P-MAPA, 1 ng/mL of rhIL-12 or their association for 48 h. In addition, P-MAPA and IL-12 were added to the lowest dose of PTX (0.625 µM) to confirm chemosensitivity. After the treatment period, cells were trypsinized and centrifuged (Centrifuge 5804 R, Eppendorf, Hamburg, Germany)) for 10 min at 1200 rpm for culture medium removal. The cell pellet was washed twice with phosphate-buffered saline (PBS) and centrifuged at 10,000 rpm for 30 s, followed by resuspension with 100 µL Annexin V binding buffer and incubation with Annexin V and propidium iodide (PI) for 15 min in the dark at room temperature. After the incubation, the prepared cells were then analyzed by flow cytometry in a FACSCantoTMII with FACSDiva (BD Biosciences, Clontech, CA, USA) software. The flow cytometric results were analyzed by the FlowJo software (vX.10.6 version, Tree Stars Inc., Ashland, OR, USA).

### 4.5. Cell Cycle Determination by PI Staining

The cell cycle stages (G0/G1, S, and G2/M) were performed by flow cytometry analysis through the DNA content measurement of nuclei stained with PI dye. After all the treatments, the SKOV-3 cells (1 × 10^5^) were trypsinized, washed with PBS, and centrifuged for 5 min at 1500 rpm. After the cells were fixed in 70% cold ethanol at 4 °C for 1 h, they were incubated with PI staining solution of 50 µg/mL PI and 10 mg/mL RNase A for 1 h at room temperature in a dark room. Flow cytometry was performed in a FACSCantoTMII with FACSDiva (BD Biosciences, Clontech, CA, USA) software to analyze DNA content. The relative ratios of cells in the G0/G1, S, and G2/M phases were calculated using FlowJo software (vX.10.6 version, Tree Stars Inc.).

### 4.6. Wound-Healing Assay

The wound-healing method was performed to verify the effects of P-MAPA and rhIL-12 alone or in combination on cell migration capacity. Briefly, 3 × 10^5^ SKOV-3 cells were placed in 6-well plates with a serum-free culture medium for cell starvation. When the cells reached high confluence, a wound was created through the confluent cell monolayer using an angled tip at 45°, and the SKOV-3 cells were then immediately treated with the pre-determined doses of P-MAPA, rhIL-12, or both, diluted in 2 mL of complete RPMI 1640 medium. Images were obtained at 0 h, 6 h, 12 h, 24 h, 36 h, and 48 h until the wound closure. Migration area (%) was measured using Image-J software. All experiments were analyzed in biological and technical triplicate.

### 4.7. Cell Migration Using Transwell Insert

To evaluate the migratory potential of the cells, 1 × 10^4^ SKOV-3 cells were seeded in triplicates into the upper chambers of 24-well ThinCert™ cell culture inserts (GBO, Americana, SP, Brazil) with PVDF filters (8.0 µm pore size) containing the corresponding treatment diluted in serum free RPMI 1640 medium. In the lower chamber, RPMI supplemented with 10% FBS was added as a chemotactic factor. After the plates were incubated at 37 °C and 5% CO_2_ for 24 h, the cells in the upper chamber were gently removed with a cotton swab. The cells that had migrated to the lower surface of the insert through the 8.0 µm pore were fixed in methanol for 8 min and stained with hematoxylin for 45 s. The migrated cells were photographed with 20× objectives under an inverted microscope (ZeissAxiovert^®^, Germany). Four non-overlapped images were randomly analyzed for each well and the migrated cells were counted using Image J software.

### 4.8. Invasion Assay

The invasion assay was performed using a 24-well ThinCert™ cell culture insert (GBO, Americana, SP, Brazil) with PVDF filters (8.0 µm pore size). Briefly, 24-well plates were previously coated with Geltrex^®^ (ThermoFisher, Waltham, MA, USA), which mimics the biological basement membrane matrix, and 1 × 10^4^ cells were placed in the upper chamber with a serum-free culture medium containing the treatments. After 24 h, the invasive potential was determined by the amount of cells capable of crossing the barrier when chemotactically attracted by the RPMI 1640 medium supplemented with 10% FBS in the lower chamber. The protocol of cell fixing and staining was the same described for cell migration. Finally, four non-overlapped images were randomly analyzed per well and the invasive cells were counted using Image J software.

### 4.9. Immunofluorescence Assay

After the SKOV-3 cells were seeded in coverslips and treated with P-MAPA, rhIL-12, or their association for 48 h, they were fixed in methanol for 8 min at room temperature and then washed in sterile ice-cold PBS. To avoid nonspecific bindings, a blocking solution containing 3% (*v*/*v*) bovine serum albumin (BSA) was added for 1 h. Then, cells were incubated overnight with rabbit polyclonal anti-TLR2 and anti-TLR4 antibodies diluted 1:200 (Abcam, Cambridge, MA) for 4 h, followed by post incubation with secondary polyclonal anti-IgG conjugated to FITC (1:200 dilution in 1% BSA, Santa Cruz Biotechnology, Inc., CA) for 1 h. After the reactions, 4,6-diamidino-2-phenylindole (DAPI; Sigma, St Louis, MO) was used for nuclei staining. Positive staining was analyzed using a confocal fluorescence microscope Zeiss Axiophot II (Carl Zeiss, Oberköchen, Germany) at different magnification (excitation filter 590 nm, emission filter 650 nm) and for DAPI staining (excitation filter 365 nm, emission filter 485 nm). The relative fluorescence in FITC images was calculated using Image J software.

### 4.10. Western Blot Analysis

After the treatments, five replicates of each experiment were used. After being washed with cold PBS, 1 × 10^6^ SKOV-3 cells were added to radioimmunoprecipitation assay buffer (RIPA) lysis buffer containing protease inhibitors and rapidly frozen for 24 h. Under constant agitation for 30 min at 4 °C, the cells were resuspended and transferred to 1.5 mL tubes and centrifuged for 20 min at 12,000 rpm. Protein quantification was performed using a Bradford assay, and the same amount of protein (40 µg) was solubilized in 1.5×Laemmli buffer and then used for 4–20% SDS-PAGE (Bio-Rad Laboratories, Hercules, CA, USA). After electrophoresis was carried out on tris-glycine running buffer system (120 V for 2 h), the proteins were electro-transferred (350 mA) to nitrocellulose membranes, and then blocked with 3% BSA in tris-buffered saline plus tween 20 (TBS-T) solution for 1 h. Afterward, the proteins were incubated with respective primary antibodies (1:500; Abcam, Cambridge, UK) at 4 °C overnight: anti-TLR2, anti-TLR4, anti-MyD88, anti-NF-kB p65, anti-TRIF, anti-IRF3. Subsequently, the membranes were washed three times and then incubated for 90 min with specific secondary antibodies (Sigma-Aldrich, St. Louis, MO, USA) diluted 1:20,000 in 1% BSA. After sequential washes, positive reactions were performed using ECL kit (Thermo Fisher Scientific, MA). All of the blots were calculated using individual samples obtained from three replicates/group and were represented as the mean optical density (band intensity/housekeeping protein). β-actin was used as the endogenous control.

### 4.11. Cytokine and Chemokine Assay

Levels of different cytokines and chemokines were determined in both supernatants and cell homogenates using a MilliPlex^®^ Map Kit (EMD Millipore, Darmstadt, Germany) with a standard 20-plex detection kit according to the manufacturer’s protocols. The human cytokine/chemokine Panel I kit (cat. no. HCYTOMAG-60K) included the following analytes: interferon-gamma (IFN-γ), interleukin (IL)-1β, IL-2, IL-3, IL-4, IL-6, IL-7, IL-8, IL-9, IL- 10, IL-12, IL-13, IL-15, IL-17, IP-10, macrophage-derived chemokine (MDC/CCL22), monocyte chemotactic protein-1 (MCP-1), macrophage inflammatory protein-1 (MIP)-1α, MIP-1β, and regulated upon activation normal T cell expressed and secreted (RANTES/CCL5). The concentrations were ranged between 0 and the lowest detectable level in each assay before log transformation. The analyses combined fluorescent cytometry and ELISA technology, so that each magnetic bead was added to a specific anti-cytokine to achieve a specific binding. For this experiment, the levels of analytes varied from 0.4 to 3500 pg/mL and the intra-assay CV was < 10%, inter-assay CV < 15%. No cross-reactivity was observed among cytokines and other molecules. The fluorescence intensity was measured using the MAGPIX system (Luminex Corporation, Austin, TX, USA).

### 4.12. Statistical Analysis

All data were evaluated using the analysis of variance (ANOVA) and presented as the mean ± standard deviation (SD). Significant results were then compared by Tukey or Newman-Keuls *post hoc* tests, and statistical significance was set at *p* < 0.05 for all analyses. Data were analyzed and constructed using GraphPad Prism 5.0 scientific graphing software (GraphPad Software, San Diego, CA, USA).

## 5. Conclusions

In summary, we demonstrated that P-MAPA in association with IL-12, both considered potent immunomodulatory agents, exhibited anti-cancer activities on human SKOV-3 cells. Although P-MAPA combined with IL-12 promotes a reduction in cell viability and cell migration, P-MAPA alone reduces the invasion capacity and enhances apoptosis in the presence of PTX. Similarly to either P-MAPA or IL-12 alone, the combinatory therapy induced the downregulation of TLR-downstream molecules involved with inflammation, which may result in protection against chemoresistance; these effects seem to be associated with TLR2 suppression rather than TLR4 signaling. P-MAPA alone stimulated the secretion of pro- and anti-inflammatory mediators, and its association with IL-12 increased the production of IL-4, -8, and MIP-1α by the SKOV-3 cells; this may promote changes in the immune responsiveness of the OC microenvironment. In addition to the effect on OC-infiltrated immune cells, these immunotherapies might provide a sustained opportunity for a novel combined strategy against malignant OC cells.

## Supplementary Materials

The following are available online. Figure S1: MTT assay was tested with different cell densities to find the most appropriate strategy for cell counting. SKOV-3 cells at density of 1 × 10^3^ were representative to determine cell viability; Table S1: Multiplex assay of the cytokines and chemokines (pg/mL) in the supernatant of cell culture; Table S2: Multiplex assay of the cytokines and chemokines (pg/mL) in the SKOV-3 cells.

## Abbreviations

BSA	Bovine serum albumin
CEEA	Ethical Committee of the Institute of Bioscience/UNESP
CEMIB	Multidisciplinary Center for Biological Investigation
DAB	Diaminobenzidine
DAPI	6-diamidino-2-phenylindole
DCs	Dendritic cells
FITC	Fluorescein Isothiocyanate
HRP-conjugated	Horseradish peroxidase-conjugated
H	Hematoxylin
IFN	Interferon
IFN-γ	Interferon gamma
IL-6	Interleukin 6
IL-8	Interleukin 8
IRF3	Interferon regulatory factor 3
MyD88	Myeloid differentiation factor 88
NF-kB p65	Nuclear factor kappa B subunit p65
NK	natural killer cells
OC	ovarian cancer
PBS	phosphate-buffered saline
P-MAPA	Protein aggregate magnesium-ammonium phospholinoleate-palmitoleate anhydride
PTX	paclitaxel
RIPA	Radioimmunoprecipitation assay buffer
SDS-PAGE	Sodium dodecyl sulphate-polyacrylamide gel electrophoresis
TBS-T	Tris-Buffered Saline plus Tween 20
CD4 + T	CD4-positive T cells
CD8 + T	CD8-positive T cells
Th1	T helper 1
TLR (s)	Toll-like receptor (s)
TLR2	Toll-like receptor 2
TLR4	Toll-like receptor 4
TRIF	TIR domain-containing adaptor inducing interferon-beta
