# Supplementary material for: P-MAPA and Interleukin-12 Reduce Cell Migration/Invasion and Attenuate the Toll-Like Receptor-Mediated Inflammatory Response in Ovarian Cancer SKOV-3 Cells: A Preliminary Study
Source: Molecules. 2019 Dec 18;25(1):5. doi: 10.3390/molecules25010005 (PMC6982916; doi:10.3390/molecules25010005)
Supplement: Supplementary file 1 [file molecules-25-00005-s001.pdf]

P-MAPA and interleukin-12 reduce cell migration/invasion and attenuate the toll-like receptor-mediated inflammatory response in ovarian cancer SKOV-3 cells

Luiz Antonio Lupi[1], Flávia Karina Delella[2], Maira Smaniotto Cucielo[1], Graziela Gorete Romagnoli[3], Ramon Kaneno[3], Iseu da Silva Nunes[4], Raquel Fantin Domeniconi[1], Marcelo Martinez[5], Francisco Eduardo Martinez[1], Wagner José Fávaro[6], Luiz Gustavo de Almeida Chuffa[1,*]

[1] Department of Anatomy, [2]Department of Morphology, [3]Department of Microbiology and Immunology, UNESP - São Paulo State University, Institute of Biosciences, Botucatu, São Paulo, Brazil.

[4]Farmabrasilis R&D Division, Campinas, SP, Brazil.

[5]Department of Morphology and Pathology, Federal University of São Carlos, SP, Brazil.

[6]Department of Structural and Functional Biology, UNICAMP - University of Campinas

Supplementary Figure. MTT assay was tested with different cell densities to find the most appropriate strategy for cell counting. SKOV-3 cells at density of 1 x 10^3 were representative to determine cell viability.

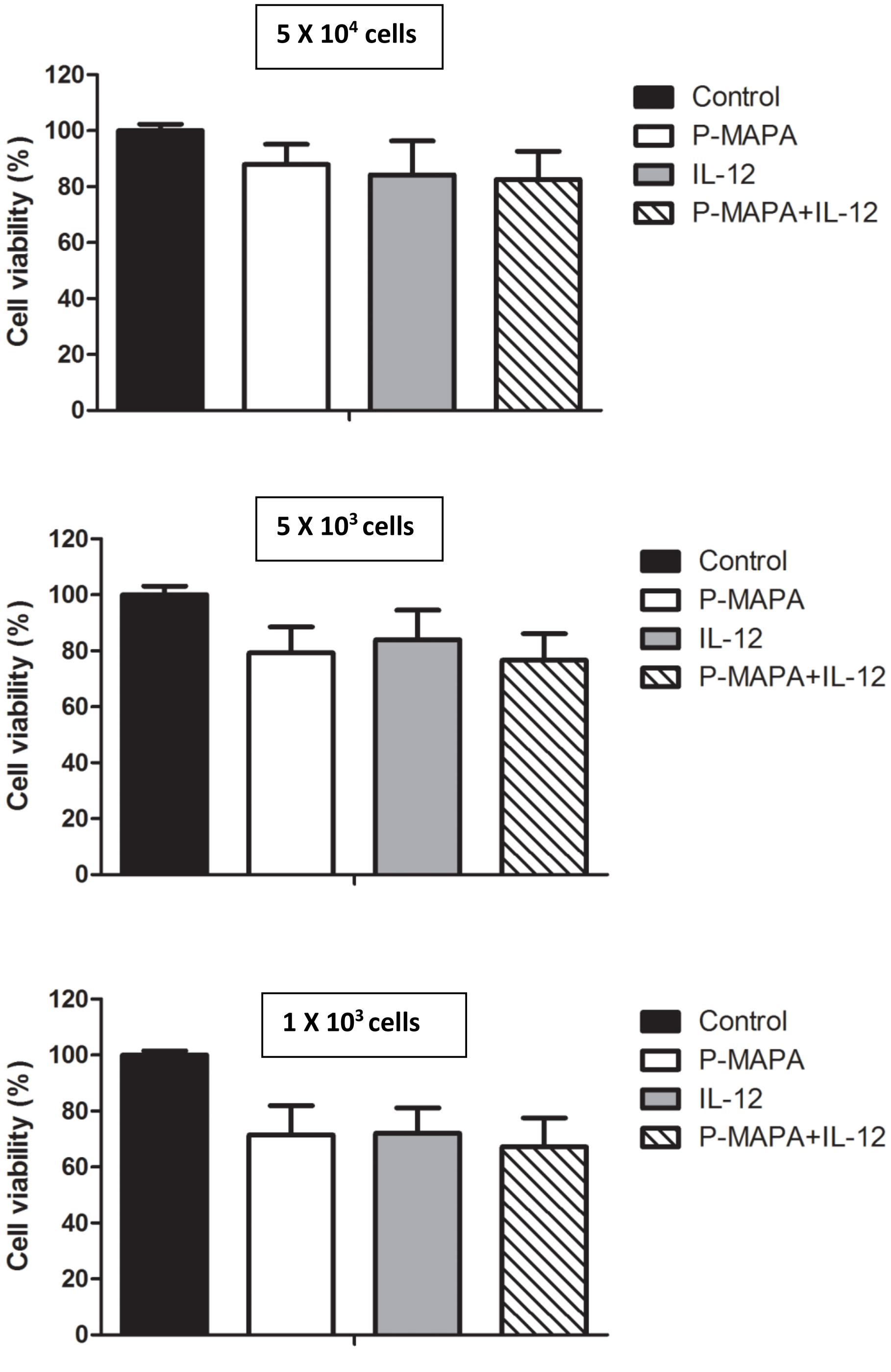

Table 1. Multiplex assay of the cytokines and chemokines (pg/mL) in the supernatant of cell culture.

Supernatant samples

Analytes	Groups				
	p	Control	P-MAPA	IL-12	P-MAPA+IL-12
IL-1β	0.57	0.95±0.03	1.00±0.04	0.99±0.08	0.90±0.05
IL-2	0.93	1.25±0.11	1.27±0.08	1.20±0.02	1.24±0.07
IL-4	***0.09***	11.12±1.43	15.42±3.20	11.34±1.01	8.16±1.23
IL-6	0.25	711.96±21.78	742.56±9.77	713.43±21.70	773.01±34.77
IL-7	***0.07***	7.63±0.63	10.38±1.20	8.02±0.51	8.02±0.51
IL-8	0.93	2425.17±212.67	2564.33±270.22	2476.67±230.15	2613.50±134.71
IL-13	0.42	5.56±0.80	7.57±1.74	5.13±0.82	5.35±0.90
IL-15	0.10	7.21±0.29	7.10±0.23	6.67±0.18	7.71±0.37
IL-17	0.29	1.13±0.028	1.22±0.04	1.16±0.03	1.16±0.04
IP-10	0.59	31.85±0.61	32.03±0.99	33.15±0.73	32.72±0.61
MCP-1	0.21	5.19±0.76	4.23±0.47	3.88±0.17	3.94±0.22
MIP-1α	0.34	2.36±0.06	2.46±0.08	2.33±0.04	2.33±0.03
MIP-1β	***0.06***	2.84±0.10	3.31±0.25	2.85±0.14	2.72±0.05

No significant values are presented for these analytes. The results are means ± SD of 3 biological replicates. *One-Way ANOVA.*

Table 2. Multiplex assay of the cytokines and chemokines (pg/mL) in the SKOV-3 cells.

Cell samples

Analytes	Groups				
	p	Control	P-MAPA	IL-12	P-MAPA+IL-12
IFN-γ	0.43	1.793±0.095	2.178±0.297	2.185±0.166	2.250±0.221
IL-1β	0.23	1.047±0.052	1.268±0.091	1.303±0.086	1.285±0.131
IL-2	0.52	1.183±0.073	1.278±0.110	1.320±0.091	1.430±0.165
IL-3	0.17	0.507±0.009	0.552±0.039	0.585±0.031	0.615±0.043
IL-6	0.86	99.333±7.386	106.290±7.668	102.955±5.242	110.415±14.494
IL-7	0.89	55.663±1.698	57.483±2.478	54.293±2.034	55.865±4.411
IL-9	0.88	72.683±10.873	78.472±13.781	75.458±5.267	67.703±5.772
IL-10	0.20	0.973±0.031	1.135±0.140	1.130±0.046	1.250±0.077
IL-12	0.22	3.113±0.200	4.165±0.583	4.308±0.439	4.662±0.678
IL-13	0.98	38.013±1.177	38.987±2.108	37.520±3.027	37.835±3.933
IL-15	0.29	54.893±3.757	63.215±5.637	67.510±6.760	76.310±11.575
IL-17	0.48	1.633±0.051	1.728±0.098	1.703±0.052	1.823±0.113
IP-10	0.48	34.030±0.349	34.105±1.365	33.590±1.477	37.765±3.623
MCP-1	0.75	2.927±0.033	3.035±0.347	3.183±0.263	3.288±0.244
MDC	0.13	2.993±0.098	3.665±0.425	4.053±0.363	3.885±0.246
MIP-1β	***0.08***	11.680±0.208	13.063±2.580	10.293±1.858	6.545±1.069
RANTES	0.21	8.597±0.724	9.580±1.646	9.800±1.087	6.590±0.700

No significant values are presented for these analytes. The results are means ± SD of 3 biological replicates. *One-Way ANOVA.*
